# Anticoagulation for left ventricular thrombi secondary to COVID—Is 3 months too long?

**DOI:** 10.1002/ccr3.5950

**Published:** 2022-06-19

**Authors:** Rimmy Garg, Amitoj Sachdeva, Juan Del Cid Fratti, Samuel Mortoti

**Affiliations:** ^1^ University of Illinois College of Medicine Peoria Peoria Illinois USA; ^2^ OSF Healthcare Cardiovascular Institute Peoria Illinois USA

**Keywords:** anticoagulation, COVID, left ventricle thrombi, STEMI

## Abstract

The length of anticoagulation for thrombotic events related to COVID‐19 is unknown. We present a patient with COVID‐19 complicated by a thrombotic anterior STEMI and multiple left ventricular (LV) thrombi that resolved after 8 weeks of anticoagulation. We suggest a shorter length of anticoagulation with COVID‐19‐related LV thrombus.

## Graphical abstract

Given the resolution of LV thrombi in our patient with COVID‐19 within 2 months, we suggest a shorter length of anticoagulation for such cases guided by echocardiographic imaging. However, prospective data regarding this matter are still needed.

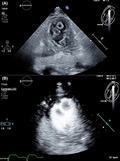

## 1 INTRODUCTION

COVID‐19 has been associated with venous and arterial thromboembolic disease likely due to potent local and systemic cytokine production with subsequent platelet activation, thrombin stimulation, and fibrin deposition.^1^ Such thromboembolic disease is treated with anticoagulation, but there are no guidelines to direct the types and duration of oral anticoagulation (OAC). We present a case of COVID‐associated acute coronary syndrome (ACS) and left ventricular (LV) thrombi with resolution of thrombi within 2 months of warfarin initiation.

## 2 CASE PRESENTATION

A 62‐year‐old female patient with a past medical history of hypertension, hyperlipidemia, and tobacco use presented with left‐sided chest pain with radiation to the left arm that started the night prior to admission. She was recently diagnosed with mild COVID‐19 infection 2 weeks earlier and was treated conservatively as an outpatient. Neither she nor her family has any history of coronary artery disease, heart failure, or any arrythmias. She was found to be tachycardic but with a regular rhythm and an otherwise normal physical examination.

Laboratory data were notable for an elevated troponin of 31.2 ng/ml (reference: <0.028 ng/ml) and elevated aspartate aminotransferase of 177 U/L. Electrolytes and an arterial blood gas values were within normal range.

**FIGURE 1:**
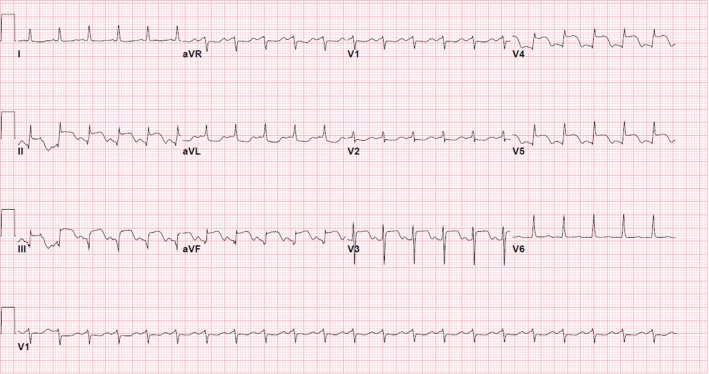
EKG demonstrating ST elevations in leads II, III, avF, and V3–V5 with ST depression in aVL

ECG demonstrated ST elevations in leads II, III, aVF, and V3–V5 with ST depression in aVL (Figure 1). With a diagnosis of anterior and inferior myocardial infarction, patient was taken emergently for a left heart catheterization (LHC).

**FIGURE 2:**
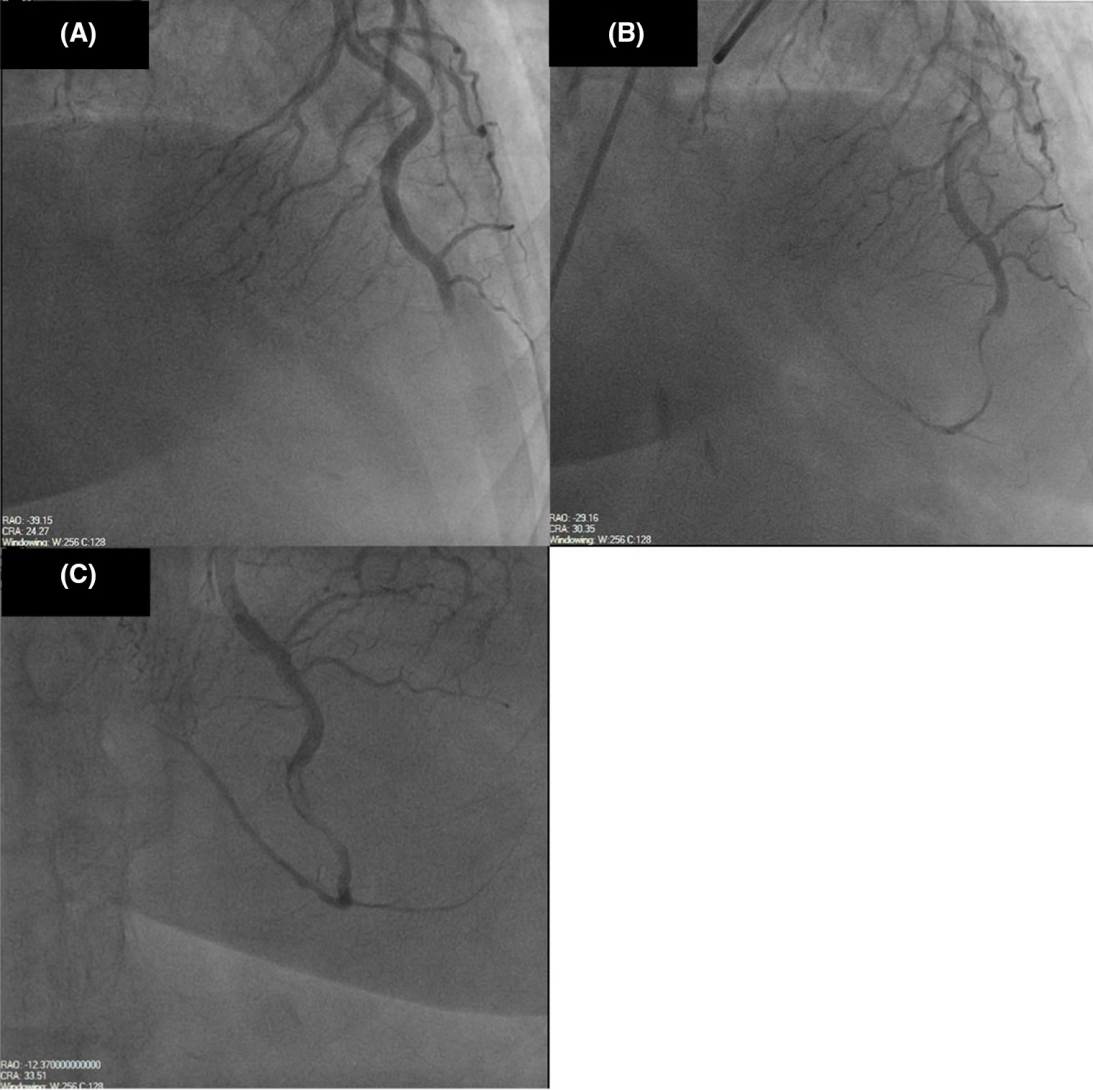
(A) Thrombotic occlusion of the proximal subsection of the distal LAD with evidence of organized thrombus. (B) Occluded LAD after percutaneous coronary intervention (PCI) with restoration of TIMI 2 flow. (C) Persistently occluded LAD 2 days after PCI. LAD, Left anterior descending

**FIGURE 3:**
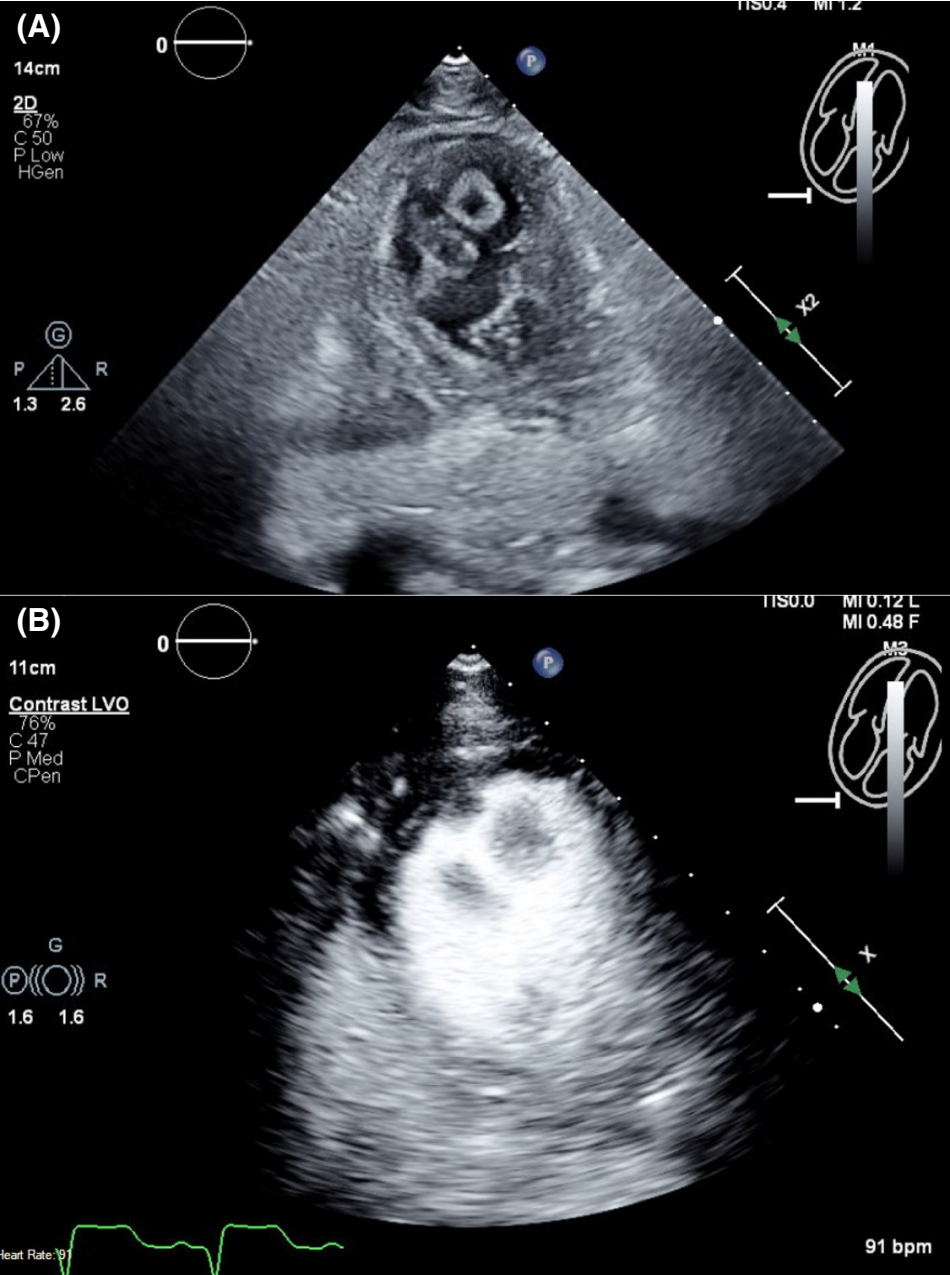
(A) TTE without contrast at the off‐axis apical 4 chamber view demonstrating multiple LV thrombi. (B) TTE with contrast at the off‐axis apical 4 chamber view demonstrating multiple LV thrombi. TTE, Transthoracic echocardiogram; LV, left ventricular

Left heart catheterization showed a thrombotic occlusion of the proximal subsection of the distal left anterior descending (LAD) coronary artery with evidence of organized thrombus, judged by the difficulty in passing a wire across (Figure 2A). Multiple balloon dilatations and multiple rounds of aspiration with a penumbra catheter were attempted, and intracoronary eptifibatide was administered with the restoration of TIMI 2 flow (Figure 2B, Video S2). Given the presence of organized clot, decision was to treat medically. The patient otherwise had non‐obstructive disease of the other coronaries. A LHC was repeated 2 days later to see whether the thrombus had resolved and stent could be placed. However, there was still residual thrombus in the distal LAD, unchanged from prior study (Figure 2C). As the patient was chest pain free and hemodynamically stable, no further intervention was attempted. A transthoracic echocardiogram (TTE) was performed, demonstrating apical akinesis with LV ejection fraction of 39% by Simpson's biplane method as well as multiple large, mobile LV thrombi with a maximum size of 2 cm × 1.5 cm (Figure 3A,B, Videos [Link], [Link]).

With systolic dysfunction in the setting of STEMI, the patient was maintained on aspirin, ticagrelor, atorvastatin, metoprolol succinate, spironolactone, and losartan. She was additionally started on a heparin drip with bridge to warfarin in the setting of multiple large LV thrombi. Given the size and number of thrombi and associated increased risk of stroke, cardiac surgery was consulted for potential surgical LV thrombus evacuation. Surgical intervention was not recommended due to high risk of complications in the setting of recent ACS, and plan was to continue medical management.

It was thought that the LAD and LV thrombi were secondary to the patient's recent COVID infection. At the time of discharge, aspirin was discontinued. Ticagrelor was to be continued for a year and warfarin for at least 3 months based on the resolution of thrombi.

**FIGURE 4:**
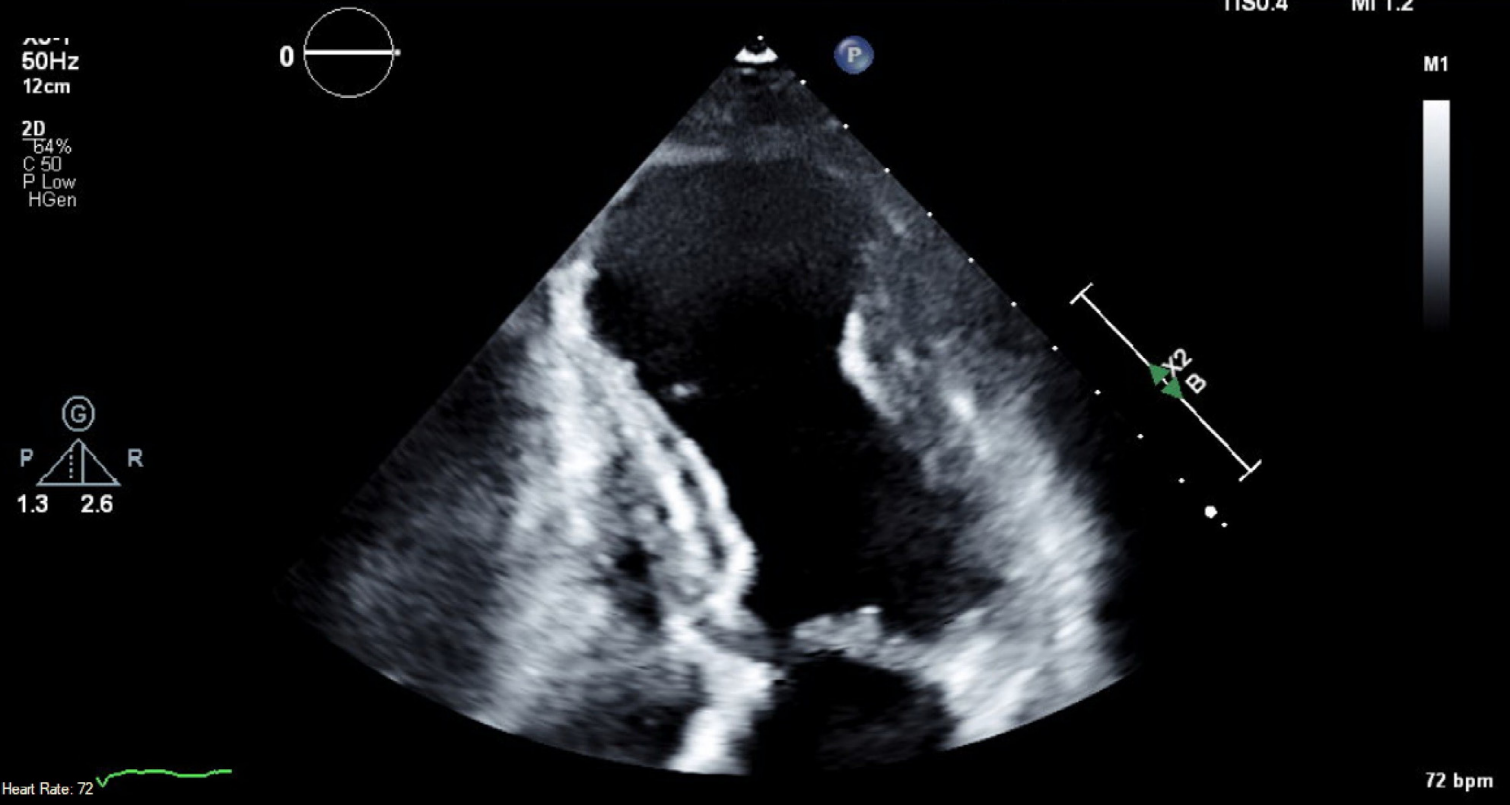
TTE without contrast at the apical 4 chamber view shows resolution of LV thrombi. TTE, Transthoracic echocardiogram; LV, left ventricular

**FIGURE 5:**
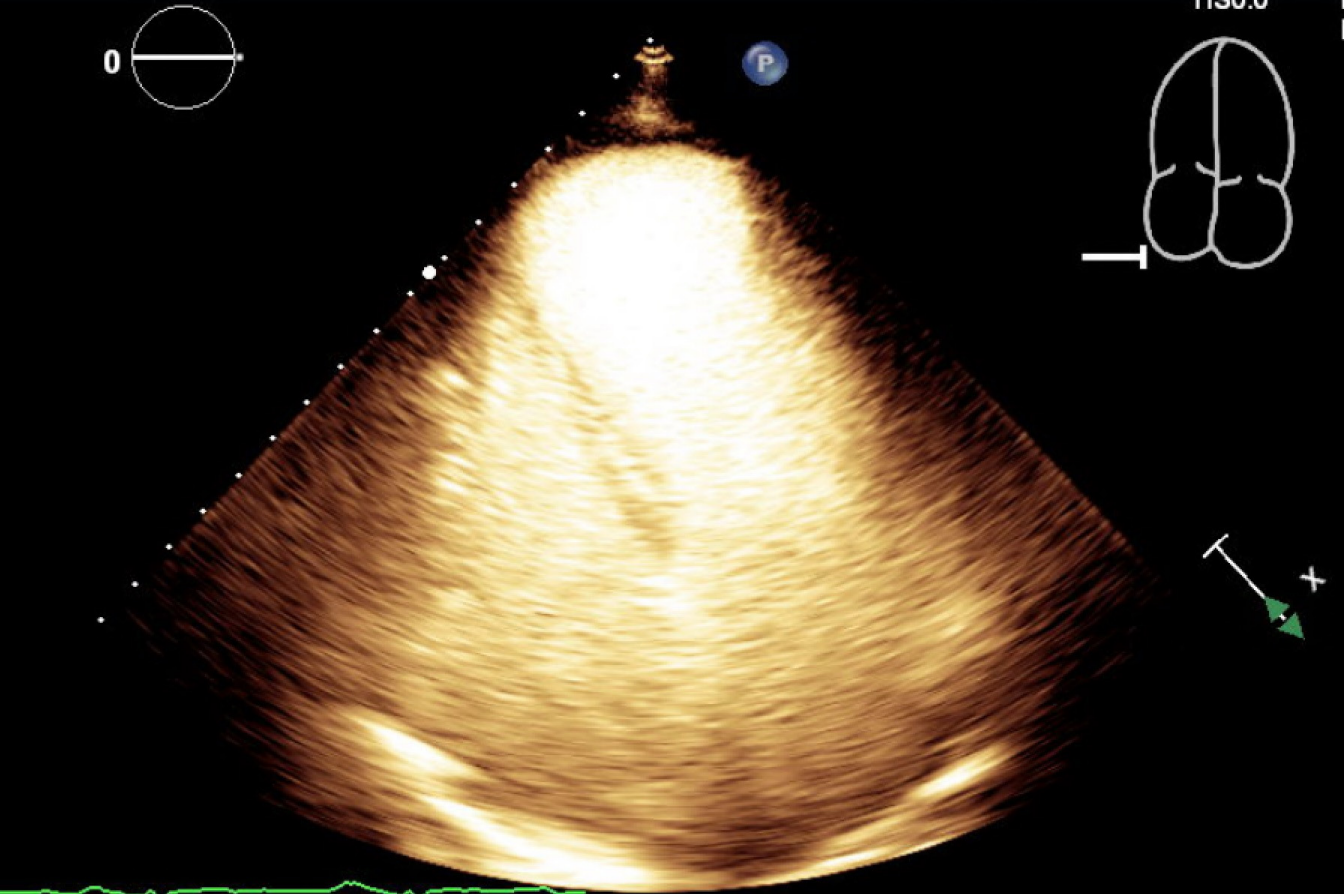
TTE with contrast at the apical 4 chamber view shows resolution of LV thrombi. TTE, Transthoracic echocardiogram; LV, left ventricular

Our patient was followed up by a cardiologist and had a repeat TTE 2 months after hospital discharge. This TTE showed that there was a large apical aneurysm of the LV but no evidence of any thrombi in the apex (Figure 4, Videos [Link], [Link]). The ejection fraction was still low around 35%. Warfarin was continued at this time due to the lack of contrast with the last TTE study (patient had refused contrast at that time), but discontinued 3.5 months later when a repeat TTE with contrast showed that the LV remained unchanged with no evidence of thrombi (Figure 5, Videos [Link], [Link]). The patient was subsequently restarted on aspirin 81 mg daily for dual antiplatelet therapy with her ticagrelor, and she remained asymptomatic on the following clinic visits.

## 3 DISCUSSION AND CONCLUSION

Cardiac manifestations of COVID‐19 ACS are due to coronary thrombosis or acute plaque rupture from systemic inflammation and catecholamine surge.^2^, ^3^ For ACS due to plaque rupture, it is recommended that dual anti‐platelet therapy and full‐dose anticoagulation be administered in the acute setting per the American College of Cardiology (ACC)/American Heart Association (AHA) and the European Society of Cardiology (ESC) guidelines.^4^ There are currently no specific guidelines on the anticoagulant used or the duration of anticoagulation in the setting of coronary thrombosis and/or LV thrombosis related to COVID‐19.

As LV thrombus is not an uncommon complication of an acute myocardial infarction, the 2013 ACC/AHA STEMI guidelines recommend OAC use in the setting of STEMI with anterior apical akinesis or dyskinesis to prevent the thrombus formation for 3 months, aiming for a lower international normalized ratio of 2.0–2.5.^5^ The 2017 ESC STEMI guidelines recommend OAC for up to 6 months with final duration guided by a repeat TTE, risk of bleeding, and need for concomitant antiplatelet therapy.^6^ The ACTION Study Group also concluded that anticoagulation for the LV thrombus for at least 3 months was associated with a lower risk of major cardiovascular events or all‐cause mortality.^7^

There have not been any large prospective or direct comparison studies looking at direct OAC (DOAC) versus warfarin for the treatment of LV thrombus. One metanalysis did show that DOACs appear to be non‐inferior to warfarin without any statistical difference in stroke or bleeding complications when treating for the LV thrombus.^8^

There have been cases that reported resolution of the LV thrombus in the setting of COVID‐19 infection prior to the 3‐month mark. One reported resolution on a 1‐month follow‐up TTE while on warfarin^9^ while another reported resolution at 10 days on low molecular weight heparin.^10^ Our patient had a large LV thrombus burden that resolved within 2 months with warfarin use.

Additionally, the risk of an LV thrombus is the highest in the first month after a myocardial infarction. In patients with a chronic LV aneurysm, the incidence of systemic emboli is extremely low, and thus, the use of long‐term OAC is not justified.^11^

As such, a shorter duration of anticoagulation under close supervision should be considered in patients with COVID‐19‐related cardioembolic/thrombotic events, guided by echocardiographic imaging. Such imaging would require a thorough sweep of the left ventricle in on‐axis and off‐axis views so as not to miss any residual thrombi. Prospective data about proper OAC regimen and duration are still needed.

## 4 AUTHOR CONTRIBUTIONS

All authors contributed equally to the preparation of this manuscript, including literature review, writing, acquiring images, and final review. All authors have read and approved the final manuscript.

## 6 CONFLICT OF INTEREST

The authors of this manuscript have no competing financial or non‐financial competing interests.

## 7 ETHICAL APPROVAL

As this was a case report with de‐identified patient information, an IRB approval was not needed.

## 8 CONSENT

The patient from this case graciously consented to the publication of this manuscript.

## Supporting information


Video S1

Video S2

Video S3

Video S4

Video S5

Video S6

Video S7

Video S8

Video S9

Video S10

Video S11

Video S12

Video S13

Video S14

Video S15

Video S16
